# An Ultra-High-Performance Liquid Chromatography Coupled with Tandem Mass Spectrometry Method with Online Solid-Phase Extraction Sample Preparation for the High-Throughput and Sensitive Determination of Ostarine in Human Urine

**DOI:** 10.3390/mps7010010

**Published:** 2024-01-23

**Authors:** Kristián Slíž, Juraj Piešťanský, Peter Mikuš

**Affiliations:** 1 Department of Pharmaceutical Analysis and Nuclear Pharmacy, Faculty of Pharmacy, Comenius University Bratislava, Odbojárov 10, 832 32 Bratislava, Slovakia; kristian.sliz@uniba.sk; 2 Toxicologic and Antidoping Centre, Faculty of Pharmacy, Comenius University Bratislava, Odbojárov 10, 832 32 Bratislava, Slovakia; 3 Department of Galenic Pharmacy, Faculty of Pharmacy, Comenius University Bratislava, Odbojárov 10, 832 32 Bratislava, Slovakia; piestansky@fpharm.uniba.sk

**Keywords:** ostarine, antidoping, online solid-phase extraction, ultra-high-performance liquid chromatography, tandem mass spectrometry

## Abstract

Ostarine is frequently misused as a selective androgen receptor modulator (SARM) in sports. Consequently, there is a pressing need for reliable and simple approaches to monitor its presence in biological systems. In this work, we developed a two-dimensional analytical method utilizing online solid-phase extraction (online-SPE) in conjunction with ultra-high-performance liquid chromatography and tandem mass spectrometry (triple quadrupole). This automated 2D separation approach is characterized by minimum manual steps in the sample preparation (only dilute-and-shoot), reflecting high sample throughput and the reliability of analytical data. It provides favorable performance parameters, including a limit of detection of 0.5 pg/mL, high accuracy (relative error = 1.6–7.5%), precision (relative standard deviation = 0.8–4.5%), and sensitivity. Additionally, it demonstrates excellent linearity (r^2^ = 0.9999) in the calibration range of 0.05 to 25 ng/mL and robustness, with no carryover effects observed. This comparative study revealed a two-decadic-order-lower LOD of the SPE-UHPLC-MS/MS method to the corresponding UHPLC-MS/MS method and the lowest one in the group of currently published LC-MS methods. The World Anti-Doping Agency screening and confirmation criteria were met through the analysis of spiked urine samples from ten healthy volunteers. Accordingly, the proposed method is suitable for routine use in antidoping laboratories.

## 1. Introduction

Ostarine is a non-steroidal, orally administered androgen receptor (AR) modulator, also known as Enobosarm or GTx-024. It was initially developed to induce anabolic effects with minimal androgenic activity while targeting specific tissues. Thus, it falls under the category of selective AR modulators (SARMs) [1]. The mechanisms responsible for its tissue selectivity are not yet fully comprehended. Nevertheless, reports suggest that ostarine can cause distinctive conformational changes when attached to AR. Different groups of coactivators and corepressors are recruited by various tissue types, resulting in the activation of different downstream signaling pathways. In addition, ostarine differentiates itself from steroidal anabolic agents (such as testosterone) as it is not subject to aromatization or 5-alpha reduction. This may differentiate its actions in tissues that require the conversion of testosterone to estradiol or dihydrotestosterone to exhibit biological effects [2].

In the clinical field, ostarine presents a novel, targeted approach to leverage the therapeutic benefits of AR modulation while avoiding virilization or estrogenic effects [1]. In the Phase 2 trials, significant improvements in lean body mass, muscle size, and the prostate’s relative preservation were observed [3,4]. Despite that, during a Phase 3 trial, consistent improvements in physical function were not exhibited [5]. Currently, ostarine is undergoing additional Phase 3 clinical evaluation as a potential treatment of AR-positive, estrogen receptor-positive, and human epidermal growth factor 2 receptor-negative metastatic breast cancer [2].

Unfortunately, in addition to its clinical advantages, ostarine has gained a reputation as a performance-enhancing drug in sports [6]. Owing to its capacity for enhancing sport performance (due to the anabolic effects discussed above), putting the health of athletes at risk [6,7,8,9,10,11,12], and violating the principles of the spirit of sport [13], the World Anti-Doping Agency (WADA) prohibited ostarine in 2008 [14]. Nevertheless, there has been a gradual increase in the misuse of ostarine during the last decade [14], peaking with 74 recorded cases in 2019 [15].

In doping control, ultra-high-performance liquid chromatography (UHPLC) coupled with tandem mass spectrometry (MS/MS) is the main analytical technique for detecting ostarine misuse. Therefore, multiple UHPLC-MS/MS methods have been developed for routine monitoring of ostarine in human urine (Supplementary Table S1) [16,17,18,19,20,21,22]. These methods included sample preparation based on a dilute-and-shoot approach [16], solid-phase extraction (SPE) [17,18], liquid–liquid extraction (LLE) [19,20,21], or dispersive liquid–liquid microextraction (DLLME) [22]. In general, the dilute-and-shoot procedure is the most straightforward, but it has several drawbacks [16,23]. For example, it lacks a pre-concentration step, which reduces sensitivity, and it can yield samples containing high levels of interfering compounds [24]. Therefore, when using this technique, it would be strongly recommended to employ an isotope-labeled internal standard [22]. While both SPE [25,26,27] and LLE [28,29] procedures effectively address the limitations of the dilute-and-shoot approach, they can be time- and resource-consuming [30]. Furthermore, the extensive manual handling of samples during these procedures can introduce additional errors in UHPLC-MS/MS analysis. DLLME offers a less work-intensive and more environmentally friendly alternative to these methods. However, it is important to note that DLLME still requires several manual steps in the sample preparation process [22].

Hence, the main aim of our work was to develop and validate an advanced UHPLC-MS/MS method with minimal sample handling that merges the benefits of SPE with a fast and simple dilute-and-shoot approach. The developed sample pretreatment technique was based on a fully automated online-SPE procedure, where an additional extraction column is attached in front of the analytical column through an appropriate valve system (which serves as an interface). The SPE-UHPLC-MS/MS method was optimized, fully validated, and compared with its 1D counterpart (UHPLC-MS/MS) for its usefulness to be used in routine antidoping control with enhanced performance parameters.

## 2. Materials and Methods

### 2.1. Chemicals, Reagents and Solutions

All chemicals and reagents were of LC-MS grade. The reference standard for ostarine was purchased from Santa Cruz Biotechnology (Heidelberg, Germany). The reference standard for andarine (used as internal standard (IS)) was purchased from Sigma-Aldrich (St. Louis, MO, USA). Methanol, water, formic acid, and acetic acid were purchased from VWR International (Radnor, PA, USA). Ammonium formate and ammonium acetate were purchased from Sigma-Aldrich (St. Louis, MO, USA).

Stock solutions were prepared by dissolving 10 mg reference standards in 10 mL methanol. Stock solutions were diluted with LC-MS water to obtain the desired concentration of working solutions. Stock and working solutions were stored at −20 °C.

### 2.2. Urine Samples

Urine samples were collected from ten volunteers (five females and five males) and stored at −80 °C until used. This was conducted according to the guidelines of the Declaration of Helsinki and approved by the Ethics Committee of the Faculty of Pharmacy at Comenius University in Bratislava (protocol code 05/2021; date of approval: 15 December 2021).

### 2.3. Instrumentation

Measurements were performed on an Agilent Infinity 1290 liquid chromatography apparatus (Agilent Technologies, Palo Alto, CA, USA) configured with a two-position, eight-port switching valve (Agilent Technologies, Palo Alto, CA, USA). The UHPLC apparatus was coupled with an Agilent 6410 triple quadrupole mass spectrometer (MS/MS) (Agilent Technologies, Palo Alto, CA, USA) equipped with an electrospray ionization (ESI) source operating in positive mode (species: [M + H]^+^). Mass Hunter software, version B.08.00 (Agilent Technologies, Palo Alto, CA, USA) was used for data acquisition and processing.

The chromatographic separation was performed as described in this paragraph. The extraction column was a Poroshell 120 EC-C18, 50 × 2.1 mm column with 1.9 µm particle size (Agilent Technologies, Santa Clara, CA, USA). The analytical column was a Kinetex Evo C18, 100 × 2.1 mm column with 2.6 µm particle size (Phenomenex, Torrance, CA, USA). The flow and composition of the mobile phase are described in Table 1. The temperature in the thermostat was constantly +40 °C. The sample injection volume was 1000 µL (in 1D approach without online-SPE: 10 µL).

MS/MS parameters were optimized by flow injection of reference standards at the 10 µg/mL concentration level. The nebulizing gas pressure was set to 60 psi, and the capillary voltage was set to 5500 V. The drying gas temperature was set to +350 °C, and the drying gas flow was set to 13 L/min. The other optimized MS/MS parameters and selected mass reaction monitoring (MRM) transitions of the diagnostic ions are presented in Supplementary Table S2.

### 2.4. Online Solid-Phase Extraction (Online-SPE)

**Figure 1:**
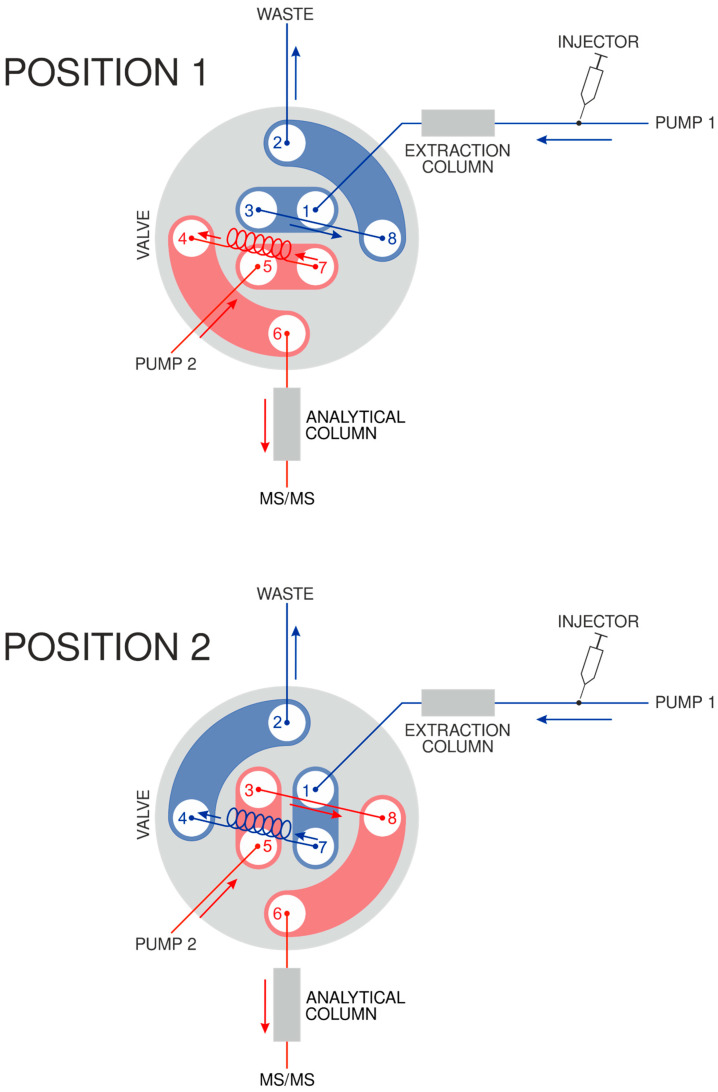
Online-SPE procedure in the SPE-UHPLC-MS/MS method. Schematic illustration of the eight-port (1–8) valve switch (position 1, position 2) system.

The online-SPE procedure developed is shown in Figure 1. It was based on a UHPLC apparatus equipped with two binary pumps, two chromatographic columns (extraction and analytical one), and a two-position eight-port switching valve. It consisted of three steps: step 1—sample loading and extraction; step 2—heart-cutting of the analyte; and step 3—separation on the analytical column.

For step 1, the valve was set to position 1 (upper scheme in Figure 1), which directs the flow from binary pump 1 through the extraction column and into a waste container. Firstly (Table 1: up to 1.00 min), a pooled human urine sample is loaded onto the extraction column. Then (Table 1: 1.01–3.50 min), the interfering compounds, such as proteins or salts, are washed into the waste. Subsequently (Table 1: 3.51–4.59 min), the percentage of organic solvent is kept high to allow the analyte to be released from the SPE column and eluted.

For step 2, the valve has been switched to position 2 (lower scheme in Figure 1), which directs the eluent from the extraction column into a 2 mL loop. This procedure (Table 1: 4.60–5.45 min) cuts the fraction of interest (i.e., containing the analyte) from the sample in a heart-cutting manner.

For step 3, the valve has been switched back to position 1 (upper scheme in Figure 1), which directs the flow from binary pump 2 through the 2 mL loop (containing the analyte) into the analytical column and finally into a triple quadrupole. Firstly (Table 1: 5.46–10.30 min), the analyte’s fraction is loaded onto the analytical column (note: the analyte takes 4 min to pass through the loop). Secondly (Table 1: 10.30–12.30 min), the solvent gradient is started, and an additional separation takes place (i.e., the analyte from the rest of the sample matrix constituents). Thirdly (Table 1: 12.30–20.00 min), the percentage of organic solvent is kept high to allow the analyte to be eluted into the MS/MS instrument.

### 2.5. Method Validation

The method was validated according to the WADA International Standard for Laboratories [31]. The following parameters were evaluated using retention times and peak areas of the quantifiers (Supplementary Table S2): (1) linearity and range, (2) limit of quantification (LOQ), (3) limit of detection (LOD), (4) accuracy and precision, (5) recovery and matrix effects, (6) carryover, and (7) selectivity and robustness.

#### 2.5.1. Linearity and Range

Calibration samples were prepared by spiking the urine samples (pooled from three volunteers and then diluted 1:9 with LC-MS water) with the working solutions of ostarine (final concentration: 0.05, 0.1, 1, 5, and 25 ng/mL; for a comparative 1D method without online-SPE: 2, 10, 20, 40, 200, and 1000 ng/mL) and IS (final concentration: 0.5 ng/mL; for a comparative 1D method without online-SPE: 5 ng/mL). The calibration samples were measured three times.

Calibration parameters (such as linear range, slope (a), standard deviation of slope (SD_a_), intercept (b), standard deviation of intercept (SD_b_), and correlation coefficient (r^2^)) were calculated in Microsoft Excel, version 2302 (Microsoft Corporation, Redmond, WA, USA).

#### 2.5.2. Limit of Quantification (LOQ)

A limit of quantification (LOQ) was determined as the lowest concentration of ostarine in the urine that can be quantified with acceptable accuracy (relative error: 80–120%) and precision (relative standard deviation < 20%).

#### 2.5.3. Limit of Detection (LOD)

A limit of detection (LOD) was determined on the spiked urine sample with a S/N ratio of 3:1.

#### 2.5.4. Accuracy and Precision

Quality control (QC) samples were prepared as described in Section 2.5.1 at three concentration levels (low, medium, and high) (final concentration: 0.25, 1.25, and 12.5 ng/mL; for a comparative 1D method without online-SPE: 5, 25, and 250 ng/mL). QC samples were measured five times per day for three consecutive days.

Accuracy was calculated as the relative error (%RE) of the corresponding intra-day (*n* = 5) and inter-day (*n* = 15) measurements.

Precision was calculated as the relative standard deviation (%RSD) of the corresponding intra-day (*n* = 5) and inter-day (*n* = 15) measurements.

#### 2.5.5. Recovery and Matrix Effects

Recovery and matrix effects were both assessed at the same concentration levels as the QC samples.

Recovery was calculated by comparing the corresponding concentrations (ng/mL) of ostarine found in the spiked urine samples with the corresponding reference values obtained from the matrix-free samples.

Matrix effects were calculated as (+) signal enhancement or (−) signal suppression by comparing the corresponding peak areas of ostarine (normalized to IS) found in the spiked urine samples with the corresponding reference values (normalized to IS) obtained from the matrix-free samples.

#### 2.5.6. Carryover

The spiked urine sample (final concentration: 2000 ng/mL of ostarine and IS) was analyzed before the pooled blank urine sample. The presence of the corresponding signals in the blank urine sample was monitored and calculated (%).

#### 2.5.7. Selectivity and Robustness

Five female and five male urine samples (of different pH and specific gravity) were spiked with working solutions of ostarine (final concentration 0.05 ng/mL (LOQ)) and IS (final concentration: 0.5 ng/mL). These samples were analyzed three times.

The parameters, namely, retention times and relative abundances of two diagnostic ions, were compared with the corresponding reference values obtained from the reference sample analyzed in the same analytical batch.

In addition, five female and five male blank urine samples were measured three times. The absence of interferences was checked.

### 2.6. WADA Criteria for Method Application

According to the WADA criteria [32], the identification of ostarine by chromatography coupled with mass spectrometry is based on the comparison of the retention time and relative abundances of at least two diagnostic ions detected in a sample with those of a reference sample analyzed in the same analytical batch.

#### 2.6.1. Chromatographic Criteria

The retention time of the chromatographic peak of ostarine in the sample must not differ by more than ±1% (note: applicable limit as the IS is not the stable isotope-labeled reference standard) from the result of the reference sample analyzed in the same analytical batch [32].

Therefore, the retention times of ostarine and IS analyzed in the samples from Section 2.5.7 were compared with the corresponding reference values obtained from the reference sample.

#### 2.6.2. Mass Spectrometric Criteria

The relative abundances of the ostarine diagnostic ions in the sample must not differ by more than ±20% (note: maximum tolerance window for relative abundances (MTWRA) applicable to the selected diagnostic ions) from the result of the reference sample analyzed in the same analytical batch. The abundance of the diagnostic ions is determined from the peak area in the integrated selected ion chromatograms [32].

Therefore, the relative abundances of ostarine and IS analyzed in the samples from Section 2.5.7 were compared with the corresponding reference values obtained from the reference sample.

## 3. Results and Discussion

### 3.1. Method Optimization

#### 3.1.1. MS/MS Parameters

The MS/MS parameters were optimized by testing various factors, such as capillary voltage (ranging from 1000 to 5000 V), drying gas temperature (ranging from 200 to 400 °C), drying gas flow (ranging from 10 to 15 L/min), and fragmentor voltage (ranging from 100 to 150 V). After evaluating the highest intensity of analytical signals as the primary criterion, the optimal values were determined to be 5500 V, 350 °C, 13 L/min, and 140 V, respectively. The collision energy (CE) was also evaluated, with the range set from 0 to 60 V. Supplementary Table S2 presents the preferred CE values that produce the diagnostic ions with the highest peak areas. Additionally, Supplementary Table S2 details the selected diagnostic ions.

#### 3.1.2. Online-SPE and UHPLC Parameters

The effects of several parameters, including mobile phase flow rate (mL/min) during sample loading, isocratic/gradient elution during analyte extraction, and mobile phase flow rate (mL/min) during analyte extraction, were investigated to optimize the online-SPE method. By analyzing the intensity of the analytical signals, the optimal values were found to be 0.5 mL/min, isocratic elution (65% methanol for 2.50 min) and 1.5 mL/min for the respective parameters (Table 2).

The effect of various mobile phase additives on detector response was also tested. The additives included 0.1% formic acid (adjusted or unadjusted with 1.0 mM ammonium formate) or 0.1% acetic acid (adjusted or unadjusted with 1.0 mM ammonium acetate). It is noteworthy that the type of additive tested had little or no effect on the retention times. Regarding the shape of the peaks, all the additives used gave satisfactory results. Hence, only the peak areas were evaluated (Table 3). Consequently, 0.1% formic acid was chosen as an optimum mobile phase additive for both online-SPE and UHPLC.

### 3.2. Method Validation

#### 3.2.1. Linearity, Range, and LOQ

**Figure 2:**
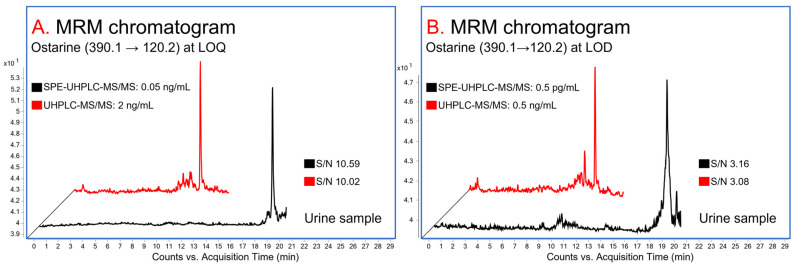
Comparison of the ostarine signals at (**A**) LOQ and (**B**) LOD concentrations obtained with (in black) or without (in red) the online-SPE procedure.

The calibration parameters are presented in Table 4. The linear relationship was determined over the range of 0.05 to 25 ng/mL with a correlation coefficient (r^2^) of 0.9999. The LOQ, representing the first point of the calibration line, was determined to be 0.05 ng/mL (Figure 2A). The LOQ obtained meets the Minimum Required Performance Levels (MRPL) for ostarine (1 ng/mL) as well as the WADA criteria (see Supplementary Tables S3 and S4 therein) [32,33].

#### 3.2.2. LOD

The LOD was determined to be 0.5 pg/mL (Figure 2B). The obtained LOD meets the WADA criteria: LOD ≤ 0.5 × MRPL [32]. It also represents the best of the previously published LODs of ostarine in urine matrices [16,17,18,19,20,21,22].

#### 3.2.3. Accuracy and Precision

The results of the analysis of the QC samples are presented in Table 5. The intra-day accuracy ranged from 92.5 to 97.9%, while the inter-day accuracy ranged from 96.7 to 98.4%. The intra-day and inter-day precision was not higher than 1.9% and 4.5%, respectively. These parameters clearly demonstrate that the minimization of sample handling due to online sample treatment results in the excellent reliability of the proposed 2D method, being favorable for its practical use.

#### 3.2.4. Recovery and Matrix Effect

The recoveries at low, medium, and high concentrations were determined to be in the range of 96.0 to 102.3% (Table 6). The corresponding matrix effect values (normalized to IS) were less than ±5%. Favorable recovery and matrix effects of the developed 2D method, as a result of an effective online sample clean-up, highlight its usefulness for analyses of variable multicomponent matrices such as human urine.

#### 3.2.5. Carryover

No carryover was observed in the blank urine sample immediately following the urine sample spiked with the high concentration of ostarine and IS. This result underlined the properly optimized physicochemical operating conditions (extractor and stationary phase type, washing, elution, equilibration, etc.) of the 2D method.

#### 3.2.6. Selectivity and Robustness

No interfering signals were observed in the blank urine samples. In the spiked urine samples (at the LOQ level, Supplementary Figure S1), the retention time (ranging from +0.1 to 0.2%) and relative abundances of two diagnostic ions (ranging from −13.9 to +6.7%) met WADA criteria (see Supplementary Tables S3 and S4 therein). Hence, the developed 2D method with enhanced orthogonality is selective and robust enough for its routine implementations in the field of multicomponent matrix analysis.

#### 3.2.7. Comparison of the Validation Parameters: SPE-UHPLC-MS/MS vs. UHPLC-MS/MS

The calibration parameters for both approaches, i.e., with (2D) and without (1D) online-SPE, are presented in Table 4. By the implementation of the online-SPE procedure, the method’s linear range was effectively moved to sub-/low ng/mL values (change by two orders of magnitude, as illustrated in Figure 2B). This enabled achieving the MRPL for ostarine of 1 ng/mL. The LOD was lowered by three orders of magnitude, from 0.5 ng/mL to 0.5 pg/mL (as illustrated in Figure 2A), as a result of a larger sample injection volume (1000 µL). This approach also improved further validation parameters, including enhancing the recovery (Table 6), minimizing matrix effects (Table 6), and enhancing the accuracy and precision (Table 5) of the measurements.

These results are largely due to the fact that the online-SPE procedure was highly effective in eliminating urine interferences (sample clean-up for enhanced selectivity and ESI efficiency) and pre-concentration of the analyte from the large sample injection volume. Moreover, fully automated sample pretreatment based on well-defined valve switching steps ensured high reproducibility of particular sample preparation steps and minimized errors originating with an external sample manipulation.

### 3.3. Method Application and Fulfilment of WADA Criteria

Following the validation process, the fitness of the developed SPE-UHPLC-MS/MS method for routine antidoping purposes was demonstrated by analyzing five spiked female and five spiked male urine samples using a “dilute-and-shoot” method.

The results of the comparison of retention time and relative abundances of two diagnostic ions are presented in Supplementary Tables S3 and S4. Briefly, (1) the difference in retention time did not exceed the maximum limit of ±1%, and (2) the difference in relative abundances of two diagnostic ions did not exceed the MTWRA of ±20%.

In addition, the S/N ratio of the spiked urine samples was evaluated (Supplementary Figure S1A), while the IS (at the concentration level of 0.5 ng/mL) was simultaneously monitored (Supplementary Figure S1B). The difference between the measured S/N values ranged from −0.63 to +1.09 (mean: 10.54; *n* = 30), indicating that the online SPE method can reliably extract ostarine from the complex urine matrices.

These results indicate the accomplishment of the WADA criteria when applying the developed SPE-UHPLC-MS/MS method for the determination of ostarine in human urine matrices. Moreover, this 2D method also fulfills the criteria of suitability for routine labs due to its high sample throughput and simple automated working protocol including whole sample preparation as well as analysis procedures.

## 4. Conclusions

The described SPE-UHPLC-MS/MS method provides a reliable analytical approach to monitor low levels of ostarine present in urine matrices. Currently, it is the most sensitive analytical method for the determination of ostarine in human urine. The incorporation of online-SPE into the analytical scheme reduces the risk of contamination and operational errors, simplifies the sample preparation process to a dilute-and-shoot procedure, and shortens the overall analytical protocol. Additionally, the use of UHPLC further improves the sample throughput. This method facilitates the efficient assessment of a notable number of samples in a brief timeframe. Each sample requires 20 min. The triple quadrupole mass spectrometer features exceptional sensitivity, making it capable of identifying very low concentrations of ostarine and, thus, spreading the time period of its detection. Moreover, with the integration of online-SPE, UHPLC, and MS/MS steps, the level of orthogonality and, by that, selectivity achieved is noticeably enhanced. This method ensures minimized potential interferences while maintaining the precise and accurate quantification of ostarine.

With respect to the practical implementation, the SPE-UHPLC-MS/MS method meets the WADA criteria and has satisfactory validation parameters. Therefore, considering the numerous benefits mentioned above, this 2D method is fit for use in routine antidoping laboratories. As a future possibility, this novel approach could also be utilized for tracking the time and concentration relationships of ostarine and its main metabolite, glucuronide-conjugated phase-II metabolite, following the administration of a single dose of ostarine to a healthy volunteer, with the advantage of a prolonged period of ostarine detection.

## Figures and Tables

**Table 1:** Timetable of the binary pump 1 and 2.

	Time (min)	A * (%)	B ** (%)	Flow (mL/min)	Process
Binary pump 1					
1	1.00	95	5	0.5	Loading
2	1.01	35	65	1.5	Extraction
3	3.50	35	65	1.5	
4	3.51	35	65	0.5	Position 2: 4.60–5.45 min
5	5.45	35	65	0.5	
6	5.46	95	5	0.5	Re-equilibrating
7	20.00	95	5	0.5	
Binary pump 2					
1	10.30	95	5	0.5	Separating
2	12.30	5	95	0.5	
3	20.00	5	95	0.5	

* solvent A: 0.1% formic acid; ** solvent B: 100% methanol.

**Table 2:** Online-SPE: optimization of the loading and washing step.

Loading	Washing			Peak Area ** (%) MRM (*m*/*z*): 390.1 → 120.2
Flow (mL/min)	Elution	B * (%)	Flow (mL/min)	
0.5	Gradient	5–65% in 2.50 min	1.5	33.3
0.5	Isocratic	65% for 2.50 min	1.5	100
0.5	Isocratic	65% for 2.50 min	0.5	65.7
1.5	Isocratic	65% for 2.50 min	1.5	85.2

* solvent B: 100% methanol; ** normalized to the highest signal.

**Table 3:** SPE-UHPLC: optimization of the mobile phase composition.

Mobile Phase Composition		Peak Area * (%) MRM (*m*/*z*): 390.1 → 120.2
Online-SPE	UHPLC	
0.1% FA	0.1% FA	100
0.1% FA	1 mM AF + 0.1% FA	90.3
1 mM AF + 0.1% FA	0.1% FA	95.6
1 mM AF + 0.1% FA	1 mM AF + 0.1% FA	85.9
0.1% AA	0.1% AA	78.1
0.1% AA	1 mM Ac + 0.1% AA	83.4
1 mM Ac + 0.1% AA	0.1% AA	80.9
1 mM Ac + 0.1% AA	1 mM Ac + 0.1% AA	82.1

FA, formic acid; AF, ammonium formate; AA, acetic acid; Ac, ammonium acetate. * normalized to the highest signal.

**Table 4:** Calibration parameters of the developed SPE-UHPLC-MS/MS method and their comparison without online-SPE.

Parameter	SPE-UHPLC-MS/MS *	UHPLC-MS/MS **
Linear range (ng/mL)	0.05 to 25	2 to 1000
Slope (a)	0.3925	0.0276
SD_a_ (*n* = 18)	0.0019	0.0001
Intercept (b)	−0.0360	−0.0427
SD_b_ (*n* = 18)	0.0194	0.0414
r^2^	0.9999	0.9999
LOQ *** (ng/mL)	0.05	2
LOD **** (ng/mL)	0.0005	0.5

SD, standard deviation; r, correlation coefficient. * injection volume: 1000 µL; ** injection volume: 10 µL; *** S/N = 10; **** S/N = 3; S/N, signal-to-noise ratio.

**Table 5:** Accuracy and precision of the developed SPE-UHPLC-MS/MS method and their comparison without online-SPE.

QC Samples	Intra-Day (*n* = 5)			Inter-Day (*n* = 15)		
c_N_ (ng/mL)	c_F_ (ng/mL)	RE (%)	RSD (%)	c_F_ (ng/mL)	RE (%)	RSD (%)
SPE-UHPLC-MS/MS *						
0.25	0.27	+7.5	1.9	0.26	+2.8	4.5
1.25	1.29	+3.5	0.8	1.27	+1.6	4.2
12.50	12.77	+2.1	0.9	12.91	+3.3	1.7
UHPLC-MS/MS **						
5	5.79	+15.9	3.9	5.59	+11.7	4.7
25	26.35	+5.4	1.7	26.34	+5.4	3.9
250	276.50	+10.6	1.7	281.96	+12.8	2.9

RE, relative error (accuracy); RSD, relative standard deviation (precision); c_N_, nominal concentration; c_F_, found concentration. * injection volume: 1000 µL; ** injection volume: 10 µL.

**Table 6:** Recovery and matrix effects of the developed SPE-UHPLC-MS/MS method and their comparison without online-SPE.

Urine/Reference Samples (*n* = 5)			
c (ng/mL)	Recovery (%)	ME without IS (%)	ME with IS (%)
SPE-UHPLC-MS/MS *			
0.25	102.3	−15.0	+3.3
1.25	98.7	−28.5	−1.4
12.50	96.0	−40.0	−4.0
UHPLC-MS/MS **			
5	81.3	−23.5	−23.7
25	76.5	−32.8	−24.7
250	75.3	−34.4	−24.8

ME, matrix effects; IS, internal standard; c, concentration. * injection volume: 1000 µL; ** injection volume: 10 µL.

## Data Availability

Data are available from the author K.S.

## Supplementary Materials

The following supporting information can be downloaded at https://www.mdpi.com/article/10.3390/mps7010010/s1. Table S1: Comparison of UHPLC-MS/MS methods for the analysis of ostarine in human urine; Table S2: Mass transitions and optimized mass spectrometer parameters applied for mass reaction monitoring (MRM); Table S3: WADA’s chromatographic identification criteria: retention time comparison; Table S4: WADA’s mass spectrometric identification criteria: relative abundance of two diagnostic ions comparison; Figure S1: Illustrative chromatograms of ten different human urine samples measured at the LOQ level of ostarine demonstrating variability of sample fractions after online-SPE heart-cut, UHPLC analytical separation, and MS/MS detection.
