# Supplementary material for: An Ultra-High-Performance Liquid Chromatography Coupled with Tandem Mass Spectrometry Method with Online Solid-Phase Extraction Sample Preparation for the High-Throughput and Sensitive Determination of Ostarine in Human Urine
Source: Methods Protoc. 2024 Jan 23;7(1):10. doi: 10.3390/mps7010010 (PMC10892632; doi:10.3390/mps7010010)
Supplement: Supplementary file 1 [file mps-07-00010-s001.zip › mps-2807345-supplementary.pdf]

methods and protocols

MDPI

Article: Supplementary Materials

An Ultra-High-Performance Liquid Chromatography Coupled with Tandem Mass Spectrometry Method with Online Solid-Phase Extraction Sample Preparation for the High-Throughput and Sensitive Determination of Ostarine in Human Urine

Kristián Slíž [1,2], Juraj Piešťanský [3] and Peter Mikuš [1,2,*]

[1] Department of Pharmaceutical Analysis and Nuclear Pharmacy, Faculty of Pharmacy, Comenius University Bratislava, Odbojárov 10, 832 32 Bratislava, Slovakia; kristian.sliz@uniba.sk
[2] Toxicologic and Antidoping Centre, Faculty of Pharmacy, Comenius University Bratislava, Odbojárov 10, 832 32 Bratislava, Slovakia
[3] Department of Galenic Pharmacy, Faculty of Pharmacy, Comenius University Bratislava, Odbojárov 10, 832 32 Bratislava, Slovakia; piestansky@fpharm.uniba.sk
* Correspondence: mikus@fpharm.uniba.sk; Tel.: +421-2-50-117-243

Contents:

Table S1. Comparison of UHPLC-MS/MS methods for the analysis of ostarine in human urine.

Table S2. Mass transitions and optimized mass spectrometer parameters applied for mass reaction monitoring (MRM).

Table S3. WADA's chromatographic identification criteria: retention time comparison.

Table S4. WADA's mass spectrometric identification criteria: relative abundance of two diagnostic ions comparison.

Figure S1. Illustrative chromatograms of ten different human urine samples measured at the LOQ level of ostarine demonstrating variability of sample fractions after online-SPE heart-cut, UHPLC analytical separation and MS/MS detection.

Table S1. Comparison of UHPLC-MS/MS methods for the analysis of ostarine in human urine.

Sample preparation	Column	Detector	LOD (ng/mL)	Ref.
Dilute-and-shoot	Nucleoshell RP18 Plus (2.0 × 50 mm, 2.7 µm)	Q-Orbitrap	< 0.1	[16]
SPE	Kinetex C18 (2.1 × 100 mm, 1.7 µm)	Orbitrap	–	[17]
Enzym. + SPE	Cortecs C18 (2.1 x 100 mm, 2.7 µm	QqQ	0.005	[18]
Enzym. + LLE	Luna Omega Polar C18 (2.1 × 100 mm, 1.6 µm)	QqQ	0.005	[19]
Enzym. + LLE	Nucleodur C18 Pyramid (2.0 × 50 mm, 1.8 µm)	Q-Orbitrap	0.02	[20]
Enzym. + LLE	Ascentis C18 (2.1 x 150 mm, 2.7 µm)	QqQ	0.1	[21]
DLLME	Kinetex C18 (2.1 × 100 mm, 1.7 µm)	QqQ	0.05	[22]
Dilute-and-shoot + Online-SPE	Kinetex EVO C18 (2.1 × 100 mm, 2.6 µm)	QqQ	0.0005	This work

LOD, limit of detection; Ref., reference; Enzym., enzymatic hydrolysis; SPE, solid-phase extraction; LLE, liquid-liquid extraction; DLLME, dispersive liquid-liquid microextraction; Online-SPE, online solid-phase extraction; Q, quadrupole; QqQ, triple quadrupole.

Table S2. Mass transitions and optimized mass spectrometer parameters applied for mass reaction monitoring (MRM).

Substance	MRM transition (*m/z*)	Species of the parent ion	Frag (V)	CE (V)
Ostarine	390.1 → 120.2 (quantifier)	$[M + H]^+$	140	36
Ostarine	390.1 → 193.2 (qualifier)	$[M + H]^+$	140	40
Andarine (IS)	442.1 → 108.1 (quantifier)	$[M + H]^+$	140	40
Andarine (IS)	442.1 → 148.1 (qualifier)	$[M + H]^+$	140	32

Frag, fragmentor voltage; CE, collision energy; IS, internal standard.

Table S3. WADA's chromatographic identification criteria: retention time comparison.

No.	Retention time: IS (min)			Retention time: ostarine (min)		
	Sample	Reference	Max. tolerated diff. ± 1%	Sample	Reference	Max. tolerated diff. ± 1%
1	18.80	18.78	+ 0.1%	18.88	18.86	+ 0.1%
2	18.80		+ 0.1%	18.88		+ 0.1%
3	18.80		+ 0.1%	18.88		+ 0.1%
4	18.80		+ 0.1%	18.88		+ 0.1%
5	18.80		+ 0.1%	18.88		+ 0.1%
6	18.80		+ 0.1%	18.88		+ 0.1%
7	18.80		+ 0.1%	18.88		+ 0.1%
8	18.80		+ 0.1%	18.88		+ 0.1%
9	18.81		+ 0.2%	18.89		+ 0.2%
10	18.81		+ 0.2%	18.89		+ 0.2%

No, sample number; IS, internal standard; Diff., difference.

Table S4. WADA's mass spectrometric identification criteria: relative abundance of two diagnostic ions comparison.

No.	Diagnostic ion (m/z)	RA (%)		Difference (%)	Tolerance window (%)
		Sample	Reference		
1	390.1 → 120.2	100	100	- 1.2	
	390.1 → 193.2	43.02	43.54		
2	390.1 → 120.2	100	100	- 10.4	
	390.1 → 193.2	39.02	43.54		
3	390.1 → 120.2	100	100	- 13.9	
	390.1 → 193.2	37.50	43.54		
4	390.1 → 120.2	100	100	- 6.5	
	390.1 → 193.2	40.70	43.54		
5	390.1 → 120.2	100	100	- 7.7	
	390.1 → 193.2	41.18	43.54		34.83–52.25 (± 20%)
6	390.1 → 120.2	100	100	- 2.4	
	390.1 → 193.2	42.5	43.54		
7	390.1 → 120.2	100	100	+ 6.7	
	390.1 → 193.2	46.46	43.54		
8	390.1 → 120.2	100	100	+ 6.7	
	390.1 → 193.2	46.46	43.54		
9	390.1 → 120.2	100	100	- 1.8	
	390.1 → 193.2	42.75	43.54		
10	390.1 → 120.2	100	100	- 5.9	
	390.1 → 193.2	40.95	43.54		

No, sample number; RA, relative abundance.

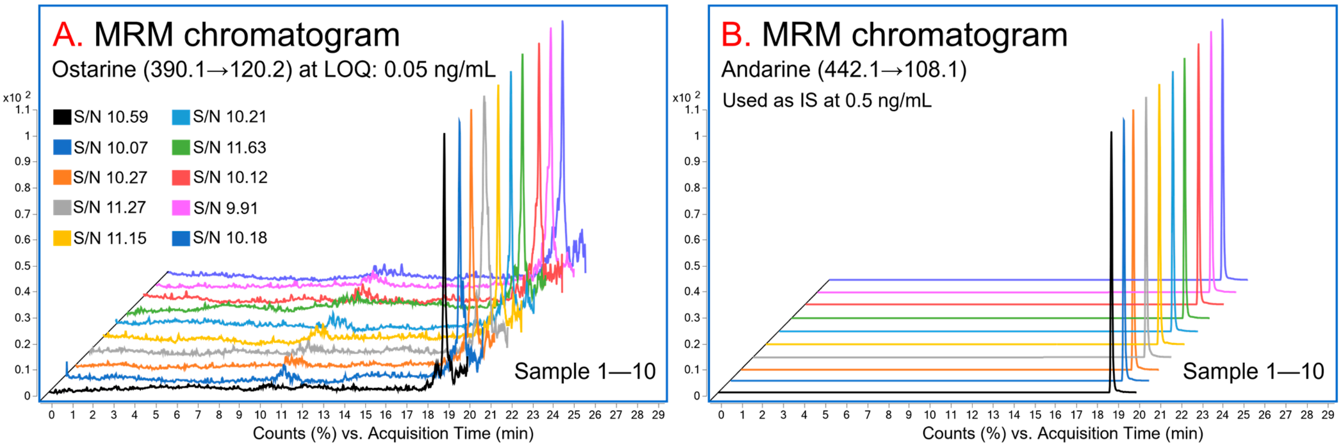

Figure S1. Illustrative chromatograms of ten different human urine samples measured at the LOQ level of ostarine demonstrating variability of sample fractions after online-SPE heart-cut, UHPLC analytical separation and MS/MS detection.
